# 3,3′-Diethylthiatricarbocyanine Iodide: A Highly Sensitive Chiroptical Reporter of DNA Helicity and Sequence

**DOI:** 10.3390/ijms12118052

**Published:** 2011-11-16

**Authors:** Jung Kyu Choi, Alessandro D’Urso, Mitch Trauernicht, Murtaza Shabbir-Hussain, Andrea E. Holmes, Milan Balaz

**Affiliations:** 1 Department of Chemistry, University of Wyoming, Laramie, WY 82071, USA; E-Mails: jchoi4@uwyo.edu (J.K.C.); adurso@unict.it (A.D.); mshabbir@uwyo.edu (M.S.-H.); 2 Doane College, 1014 Boswell, Crete, NE 68333, USA; E-Mails: mitchell.trauernicht@doane.edu (M.T.); andrea.holmes@doane.edu (A.E.H.)

**Keywords:** Cy7 cyanine dye, left-handed Z-DNA, circular dichroism, DNA recognition, sensing

## Abstract

Using UV-vis absorption and circular dichroism (CD) spectroscopies, we explored the binding interactions of 3,3′-diethylthiatricarbocyanine iodide (**Cy7**) with polynucleotides of different sequences and helicity. CD showed to be a very diagnostic tool giving different spectroscopic chiroptical signatures for all explored DNA sequences upon **Cy7** binding. **Cy7** was able to spectroscopically discriminate between the right handed B-DNA of poly(dG-dC)_2_ and its left handed Z-DNA counterpart induced by spermine or Co(III)hexamine via nearly opposite induced circular dichroic signal.

## 1. Introduction

Cyanine dyes represent an important class of chromophores due to their favorable optical properties, such as high extinction coefficients and fluorescence [1]. Their highly conjugated structure results in a small HOMO-LUMO gap and red shifted absorbance and fluorescence [2,3]. Cyanines cover a wide span of applications ranging from fluorescent biomedical imaging, labeling, and non-linear optics to light harvesting and optical storage [4–8].

**Chart 1:**
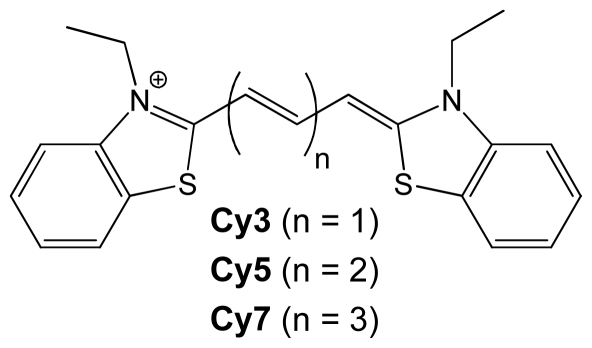
Structure of Cy3, Cy5, and Cy7.

Cyanines are achiral and thus circular dichroism (CD) silent in the absence of a chiral template like DNA. Binding of the achiral dyes to the chiral DNA helix can result in an induced circular dichroism (ICD) in the absorption spectrum of the dye (500–900 nm). An ICD signal can rise from two distinct phenomena, (a) chiral twisting of the dye in the DNA groove; or (b) an exciton coupling between two chirally oriented dyes. Since cyanines absorb in the visible region whereas nucleic acids absorb in the UV region (<300 nm), the ICD signal is free of overlaps and very diagnostic of a dye binding mode. Cy3 and Cy5 cyanine dyes have previously been reported to bind to alternating adenine-thymine oligo and polynucleotides. Cy5 assembles as parallel helical dimers in the minor groove, and exciton coupled circular dichroism (ECCD) originates from the interaction between the adjacent cyanine dimers (dimer-dimer coupling) [9–13]. However, binding of Cy5 (Cy3 was not studied) to poly(dG-dC)_2_ did not yield an ICD signal in the cyanine absorption region. The absence of an ICD was explained by the ineffective, non-coupled orientation of Cy5 upon DNA binding [13]. 3,3′-Diethylthiatricarbocyanine iodide (**Cy7**, Chart 1) contains a conjugated bridge of seven methines and has a more red shifted absorption than Cy5 (Δλ_max_ ~ 100 nm) with an absorption maxima in the NIR region (650 to 800 nm). The extended conjugated system makes **Cy7** dye more photolabile than its shorter counterparts, and long-term exposure to visible light must be avoided. Herein we report the chiroptical signature of **Cy7** binding with polynucleotides having different sequences and helical twists.

The spectroscopic recognition of DNA helicity is important but challenging [14–18]. The biological relevance of Z-DNA has been demonstrated by the discovery of transcription factors that selectively bind to Z-DNA, and thus have a direct impact on gene expression [19–22]. Z-DNA is left handed and is higher in energy than the canonical right-handed B-DNA [23,24] Thus far, porphyrins [16,18,25–28] helicines [15], and tris(phenanthroline)metal-complexes [29–31] have been used as *in vitro* Z-DNA probes. No *in vivo* molecular probes have been reported so far. In order to explore the DNA binding of **Cy7**, we have selected three polynucleotide sequences allowing us to access four DNA duplexes that differ in nucleobase sequence and helicity: (i) the B-form of poly(dA-dT)_2_; (ii) the B-form of poly(dC).poly(dG); (iii) and (iv) the B- and Z-forms of poly(dG-dC)_2_.

## 2. Results and Discussion

### 2.1. UV-vis Spectroscopy of the B-forms of poly(dA-dT)_2_, poly(dC).poly(dG), and poly(dG-dC)_2_

**Figure 1:**
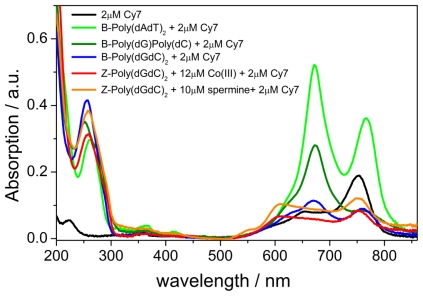
Absorption data for **Cy7** dye alone (black), and in the presence of poly(dA-dT)_2_ (light green), poly(dG).poly(dC) (green), poly(dG-dC)_2_ (blue), Co(III)-Z-poly(dG-dC)_2_ (red), and spermine-Z-poly(dG-dC)_2_ (orange). Conditions: [DNA] = 50 μM, [NaCl] = 10 mM, 5% MeOH in Na-cacodylate buffer (1mM, pH = 7.0).

The UV-vis absorption spectrum of **Cy7** in the absence of DNA showed a characteristic profile with two major bands at 755 nm (strong) and 650 nm (weak). Absorption spectra show that very diverse structural and electronic mechanisms exist when **Cy7** is bound to different forms of DNA (Figure 1). Titration of **Cy7** into a solution of alternating adenine-thymine polynucleotide poly(dA-dT)_2_ resulted in a significant increase of intensity at 650 nm and 750 nm which was accompanied by the shift of the absorption maxima to a longer wavelength. The **Cy7** (2 μM) bound to the adenine-thymine DNA (50 μM) showed a red shift from 650 nm to 670 nm (Δλ = 20 nm, 80% hyperchromicity) and from 750 nm to 760 nm (Δλ = 15 nm, 100% hyperchromicity) when compared to the DNA-free unbound dye. Similar absorption behavior was observed for the shorter cyanine dye, Cy5, where changes in absorption behavior were explained as a result of a Cy5 dimer formation [9–12]. On the other hand, addition of **Cy7** (0 to 2 μM) to a solution of non-alternating polynucleotide poly(dG).poly(dC) resulted in a decrease (40% hyperchromicity) of the 750 nm absorption band and an increase (100% hyperchromicity) of the 650 nm band. Both bands exhibited bathochromic shifts, Δλ _650_ = 20 nm and Δλ _750_ = 5 nm. Titration of Cy5 into a poly(dG-dC)_2_ has previously shown to result in hypochromicity of absorption bands without formation of a cyanine dimer. In our case, however, the increase of 650 nm absorption band together with a red shift (from 650 to 670 nm, Δλ = 20 nm, 15% hypochromicity) suggested the formation of a **Cy7** dimer upon DNA binding. The decrease of absorption (45% hypochromicity) and red shift of 750 nm band (Δλ = 20 nm) furnished additional evidence for the **Cy7** dimer formation in the presence of poly(dG-dC)_2_. The changes in UV-vis spectroscopy when **Cy7** is bound to different forms of DNA originate from structural differences of **Cy7** in the minor groove of three examined polynucleotides. Interestingly, cyanine dyes Cy3 and Cy5 were previously found to bind to poly(dG-dC)_2_ as monomers while our results suggest the formation of **Cy7** dimers in the presence of poly(dG-dC)_2_.

### 2.2. CD Spectroscopy of the B-form of poly(dA-dT)_2_

**Figure 2:**
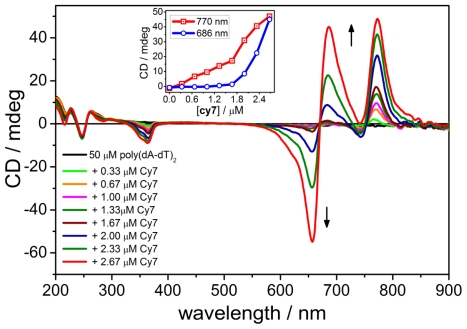
CD spectra of **Cy7** titrated to poly(dA-dT)_2_. Inset: intensity change of the CD signals at 770 nm and 686 nm as a function of the **Cy7** concentration. Conditions: [poly(dA-dT)_2_] = 50 μM, [NaCl] = 10 mM, 5% MeOH in Na-cacodylate buffer (1 mM, pH = 7.0). Titration step: [**Cy7**] = 0.33 μM.

Titrations of **Cy7** (from 0 μM to 1.66 μM, 0.33 μM increment) into a solution of poly(dA-dT)_2_ gave rise to a positive CD band at 770 nm and a negative CD band at 360 nm (Figure 2). These CD bands originated from the chiral twist of a DNA bound dye. The 770 nm CD band coincided with 770 nm absorption band corresponding to the monomeric form of the dye. Increasing the concentration of **Cy7** from 1.66 μM to 2.66 μM resulted in appearance of a bisignate CD signal with a positive CD band at 686 nm and negative band at 655 nm accompanied with an additional increase of ellipticity of the 770 nm CD band (Inset, Figure 2). This bisignate CD originated from electronic dipole-dipole exciton coupling between two neighboring cyanine dyes. The isosbestic point of the bisignate CD signal overlapped with the absorption band at 670 nm and provided additional evidence that the bisignate CD curve rose from exciton coupling involving **Cy7** dimers. The binding of **Cy7** did not disturb the secondary structure of DNA which could be seen from the nearly unchanged characteristic DNA region in the UV region of the CD spectrum.

### 2.3. CD Spectroscopy of the B-form of poly(dC).poly(dG)

**Figure 3:**
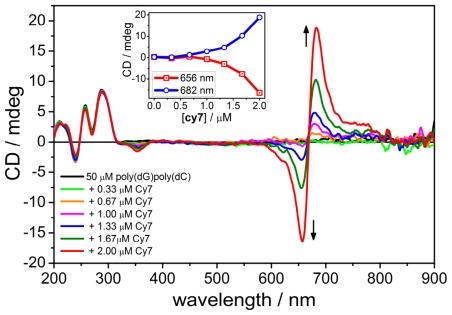
CD spectra of **Cy7** titrated to poly(dG).poly(dC). Inset: intensity change of the CD signal as a function of the **Cy7** concentration. Conditions: [poly(dG).poly(dC)] = 50 μM, [NaCl] = 10 mM, 5% MeOH + Na-cacodylate buffer (1 mM, pH = 7.0). Titration step: [**Cy7**] = 0.33 μM.

CD titration of **Cy7** to a solution of poly(dG).poly(dC) in 5% MeOH/Na-cacodylate buffer revealed a strong bisignate signal with positive Cotton effect at 680 nm and a negative Cotton effect at 655 with an isosbestic point at 668 nm (Figure 3). A small negative CD band was also observed at 350 nm. No CD band was observed at 770 nm which coincided with a very weak absorption band at that wavelength. It appears that poly(dG).poly(dC) DNA promotes the formation of chiral dimer aggregates even at low concentration of **Cy7**. Again, virtually no changes have been detected in the CD spectrum below 300 nm.

### 2.4. CD Spectroscopy of the B-form of poly(dC-dG)_2_

**Figure 4:**
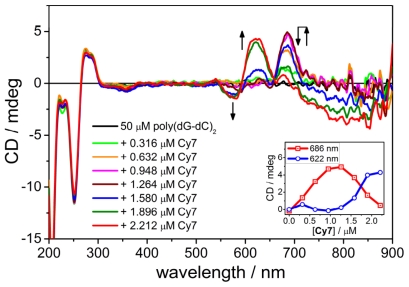
Titration of **Cy7** to poly(dG-dC)_2_. Titration step: [**Cy7**] = 0.33 μM. Inset: intensity change of the CD signals at 622 nm and 686 nm as a function of the **Cy7** concentration. Conditions: [poly(dG-dC)_2_] = 50 μM, [NaCl] = 10 mM, 5% MeOH + Na-cacodylate buffer (1 mM, pH = 7.0).

Next, we explored the binding of **Cy7** with poly(dG-dC)_2_. Stepwise addition of **Cy7** (in 0.33 μM addition steps) from 0 μM to 1.26 μM resulted in an appearance of a positive Cotton effect centered at 690 nm corresponding to a bound **Cy7** monomer (Figure 4). In addition, a very weak negative CD band was observed at 340 nm. Increasing the concentration of **Cy7** further (from 1.58 μM to 2.21 μM) yielded negative Cotton effects at 850 and 580 nm and a positive Cotton effect at 620 nm accompanied with a disappearance of the positive CD band at 690 nm (Inset, Figure 4). The observed CD spectroscopic changes originated from a rearrangement of the DNA bound **Cy7** upon addition of more dye suggesting a different DNA binding mode at low **Cy7**/DNA ratio (<1:50, *i.e.*, one dye bound for 50 DNA base pairs) and high **Cy7**/DNA ratio (>1:50).

**Figure 5:**
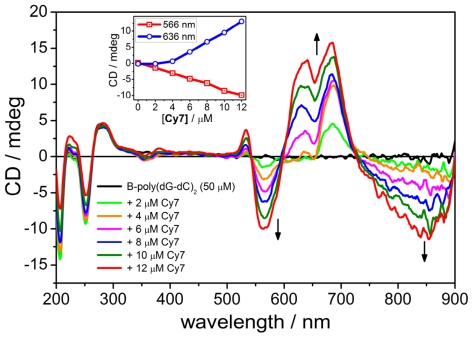
CD spectra of **Cy7** titrated to poly(dG-dC)_2_. Inset: intensity change of the CD signal as a function of the **Cy7** concentration. Titration step: [**Cy7**] = 2.0 μM. Conditions: [poly(dG-dC)_2_] = 50 μM, [NaCl] = 10 mM, 5% MeOH + Na-cacodylate buffer (1 mM, pH = 7.0).

Since the ICD signal was weak in comparison to **Cy7** binding with poly(dA-dT)_2_ or poly(dG).poly(dC) when using 0.33 μM increments, we decided to try larger additions (2 μM) to enhance the ICD signal. As can be seen in Figure 5, the first two additions of **Cy7** (2 and 4 μM) to poly(dG-dC)_2_ gave rise to a positive Cotton effect at 680 nm and a negative Cotton effect at 560 nm. Further addition of **Cy7** yielded an additional positive CD band at 640 nm, a small positive CD band at 535 nm and a broad negative CD band at 850 nm (Figure 4). It is worth noting that the previously reported shorter cyanine Cy5 dye did not yield an ICD signal when bound to poly(dG-dC)_2_ [13].

### 2.5. UV-vis and CD Spectroscopies of the Z-form of poly(dC-G)_2_

We used poly(dG-dC)_2_ as a tunable B- to Z-DNA scaffold to access DNA sequences having identical nucleotide composition but different helicity [25]. The fully protonated tetraamine spermine (H_3_N^+^-(CH_2_)_3_-^+^NH_2_-(CH_2_)_4_-^+^NH_2_-(CH_2_)_3_-^+^NH_3_) and cobalt(III) hexaamine were employed as micromolar inducers of the Z-DNA conformation [32]. Spermine-Z-DNA was induced at 60 °C using 10 μM spermine, then slowly cooled to RT (1 °C/min) while Co(III)-Z-form was induced with 12 μM at room temperature [33]. We used two different Z-DNA inducers to investigate the effect of the inducer as an integral part of the Z-DNA structure upon cyanine binding. Successful formation of Z-DNA was confirmed by CD spectroscopy where the spectral region below 300 nm revealed a spectral signature characteristic of left-handed Z-DNA, *i.e.*, negative CD bands at 290 nm and 200 nm and a positive CD band at 260 nm. Since we employed different amounts of Z-DNA inducers (10 μM of spermine^4+^ *vs*. 12 μM of Co(NH_3_)_6_^3+^) the final Z-DNA solutions differed in ionic strengths.

**Figure 6:**
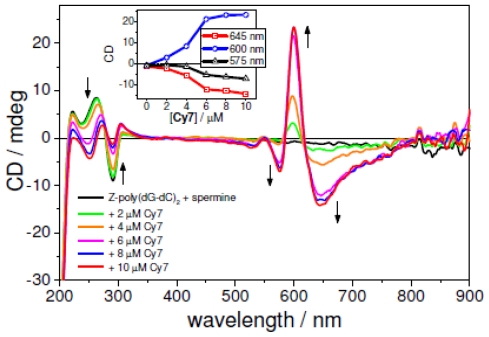
CD spectra of **Cy7** titrated to spermine induced Z-poly(dG-dC)_2_. Inset: intensity change of the CD signal as a function of the **Cy7** concentration. Conditions: [Z-poly(dG-dC)_2_] = 50 μM, [spermine] = 10 μM, [NaCl] = 10 mM, 5% MeOH + Na-cacodylate buffer (1 mM, pH = 7.0). Titration step: [**Cy7**] = 2.0 μM.

Stepwise addition of **Cy7** (0 μM to 10 μM, 2.0 μM step) to a solution of Co(III) induced Z-form of poly(dG-dC)_2_ had a distinct effect on the **Cy7** UV-vis absorption profile (Figure 1). A 50% decrease of absorbance at 750 nm without a wavelength shift has been observed. The 650 nm band exhibited 20% hypochromicity and 50 nm blue shift to 600 nm. Titration of **Cy7** to a spermine induced Z-form yielded a similar spectroscopic signature, *i.e*., a 35% hypochromicity of the 750 nm band and 20% hyperchromicity of 650 nm band accompanied with a 50 nm blue shift. Addition of **Cy7** to the spermine induced Z-poly(dG-dC)_2_ gave rise to an ICD signal at 500–800 nm with two negative Cotton effects at 645 nm and 570 nm and a positive Cotton effect at 605 nm (Figure 6). The addition of **Cy7** to spermine Z-DNA caused significant conformational changes of DNA. As seen in Figure 6, the negative CD band at 290 nm decreased dramatically upon **Cy7** addition. No such change was observed with Co(III) induced Z-DNA (Figure S2), suggesting a lower conformational stability of spermine induced Z-DNA probably caused by a binding competition between spermine and **Cy7** in the DNA minor groove.

**Figure 7:**
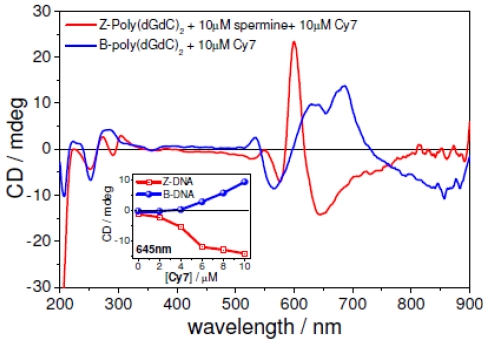
CD spectra comparison of **Cy7** bound to poly(dG-dC)_2_ (blue) and spermine induced Z-poly(dG-dC)_2_ (red). Inset: intensity change of the CD signal at 645 nm as a function of the **Cy7** concentration. Conditions: [DNA] = 50 μM, [spermine] = 10 μM, [NaCl] = 10 mM, 5% MeOH + Na-cacodylate buffer (1 mM, pH = 7.0). Titration step: [**Cy7**] = 2.0 μM.

The addition of **Cy7** to spermine induced left-handed forms of poly(dG-dC)_2_ yielded ICD spectra in the visible region with nearly opposite CD signatures when compared to the B-form of poly(dG-dC)_2_ (Figure 7). The origin of the nearly opposite CD signals was due to the dye’s opposite chiral orientation when bound to the two different DNA helical backbone. This opposite characteristic of **Cy7** was clearly seen when the ICD signal of **Cy7** bound to B-poly(dG-dC)_2_ (Figure 7, blue curve) was compared to ICD signal of the **Cy7** bound to Z-form of poly(dG-dC)_2_ induced by spermine (Figure 7, red curve) and by Co(NH_3_)_6_^3+^ (ESI, Figure S3). Therefore, **Cy7** allowed for the visualization of DNA structure in the visible spectral range which is far from any possible spectral overlap with indigenous chromophores.

## 3. Experimental Section

3,3′-Diethylthiatricarbocyanine iodide **Cy7** (3-Ethyl-2-[7-(3-ethyl-2-benzothiazolinylidene)-1,3,5-heptatrienyl]benzothiazolium iodide, DiSC2(7)) was purchased from Sigma-Aldrich. Water was obtained from a Milli-Q system with a resistivity of 18.2 MΩ·cm. DNA samples were dissolved in a sodium cacodylate buffer (1 mM, pH 7.0), annealed at 80 °C for 20 min, cooled at 1 °C/min, and kept at 4 °C. The concentration of the DNA stock solutions was quantified by UV-vis spectroscopy and is reported per base pair. The **Cy7** stock solution (*c* = 0.5 mM) was prepared in methanol, and the concentration was determined by UV-vis spectroscopy using the extinction coefficient ɛ = 2.5 × 10^5^ M^−1^·cm^−1^ at 758 nm [9,33].

CD spectra were recorded at 20 °C using a Jasco J-815 spectropolarimeter equipped with a single position Peltier temperature control system using following conditions: scanning speed 50 nm/min, data pitch 0.5 nm, DIT 2 s, and bandwidth 1 nm. UV-vis absorption spectra were collected at 20 °C using a Jasco V-600 UV-vis double beam spectrophotometer equipped with a single position Peltier temperature control system. To minimize the **Cy7** photobleaching, all titrations have been performed under reduced light and each CD spectrum was performed as a single scan. A quartz cuvette with a 1 cm path length was used for all CD and UV-vis experiments.

## 4. Conclusions

CD spectroscopy was employed to explore the chiroptical behavior of cyanine dye **Cy7** in the presence of DNA sequences having different sequences and helical twists. UV-vis absorption spectra reflected very different structural and electronic characteristics of **Cy7** when bound to different DNA forms. **Cy7** assembles onto poly(dG-dC)_2_ with a very distinct chiroptical signature, unlike its shorter cyanine counterparts Cy3 and Cy5. We showed that **Cy7** can spectroscopically discriminate between polynucleotides having different sequences using ICD signals in the visible spectroscopic region. **Cy7** also recognized and chiroptically distinguished right-handed B-DNA and left handed Z-DNA forms of poly(dG-dC)_2_ via a very diagnostic induced circular dichroism signal between 500–900 nm.

## Supplementary Material
