# Supplementary material for: 3,3′-Diethylthiatricarbocyanine Iodide: A Highly Sensitive Chiroptical Reporter of DNA Helicity and Sequence
Source: Int J Mol Sci. 2011 Nov 16;12(11):8052–62. doi: 10.3390/ijms12118052 (PMC3233455; doi:10.3390/ijms12118052)
Supplement: Supplementary file 1 [file ijms-12-08052-s001.pdf]

Supplementary Information

Jung Kyu Choi [1], Alessandro D'Urso [1,2], Mitch Trauernicht [2], Murtaza Shabbir-Hussain [1], Andrea E. Holmes [2] and Milan Balaz [1,*]

[1] Department of Chemistry, University of Wyoming, Laramie, WY 82071, USA;
E-Mails: jchoi4@uwyo.edu (J.K.C.); adurso@unict.it (A.D.); mshabbir@uwyo.edu (M.S.-H.)
[2] Doane College, 1014 Boswell, Crete, NE 68333, USA;
E-Mails: mitchell.trauernicht@doane.edu (M.T.); andrea.holmes@doane.edu (A.E.H.)

* Author to whom correspondence should be addressed; E-Mail: mbalaz@uwyo.edu;
Tel.: +1-307-766-4330; Fax: +1-307-766-2807.

Received: 21 October 2011; in revised form: 2 November 2011 / Accepted: 7 November 2011 / Published: 16 November 2011

Abstract: Using UV-vis absorption and circular dichroism (CD) spectroscopies, we explored the binding interactions of 3,3′-diethylthiatricarbocyanine iodide (**Cy7**) with polynucleotides of different sequences and helicity. CD showed to be a very diagnostic tool giving different spectroscopic chiroptical signatures for all explored DNA sequences upon **Cy7** binding. **Cy7** was able to spectroscopically discriminate between the right handed B-DNA of poly(dG-dC)$_2$ and its left handed Z-DNA counterpart induced by spermine or Co(III)hexamine via nearly opposite induced circular dichroic signal.

Keywords: Cy7 cyanine dye; left-handed Z-DNA; circular dichroism; DNA recognition; sensing

---

Figure S1. Intensity change of the CD signals at 622 nm, 770 nm, and 800 nm as a function of the **Cy7** concentration when titrated with poly(dA-dT)$_2$ (red curve with triangles)**,** poly(dG-dC)$_2$ (blue curve with squares), poly(dG).poly(dC) (green curve with spheres)**.** Titration step: [Cy7] = 2.0 μM. Conditions: [DNA] = 50 μM, 10 mM NaCl, 5% MeOH + Na-cacodylate buffer (1mM, pH = 7.0).

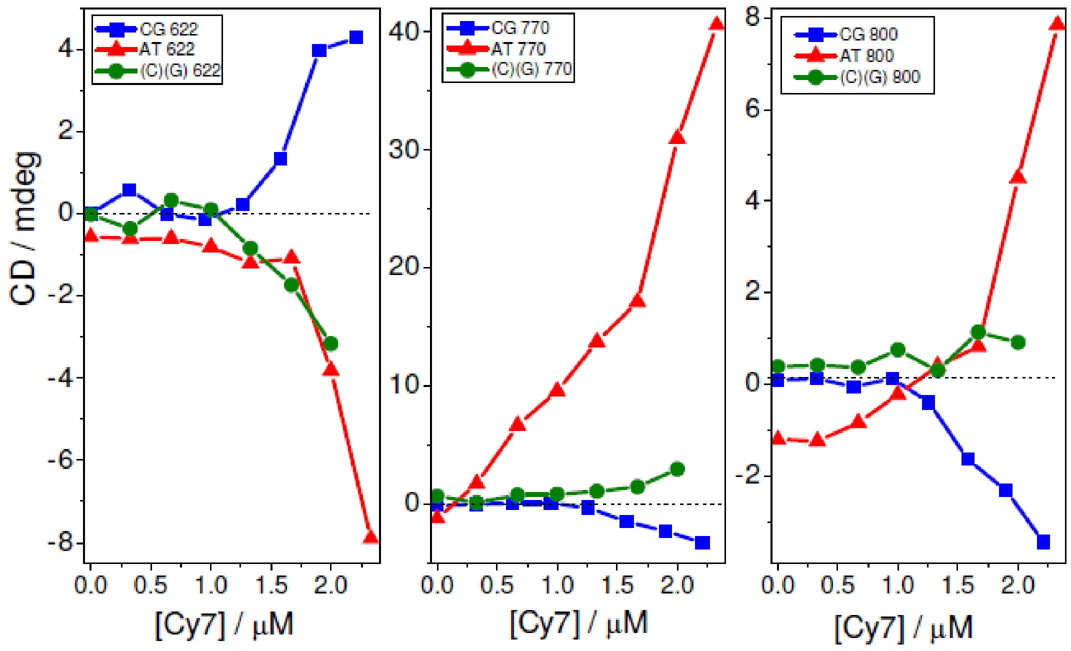

Figure S2. CD spectra of **Cy7** titrated to Co(III) induced Z-poly(dG-dC)$_2$. Titration step: [Cy7] = 2.0 μM. Conditions: [Z-poly(dG-dC)2] = 50 μM, [Co(III)] = 12 μM, [NaCl] = 10 mM, 5% MeOH + Na-cacodylate buffer (1mM, pH = 7.0).

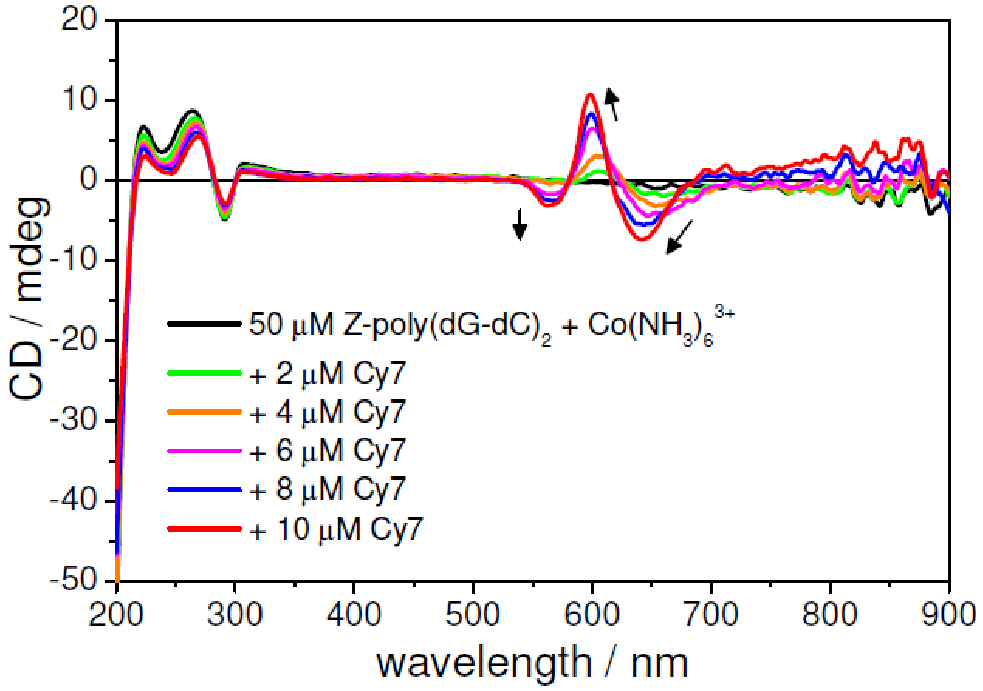

Figure S3. CD spectra comparison of **Cy7** bound to poly(dG-dC)$_2$ (blue) and Co(III) induced Z-poly(dGdC)$_2$ (red). Inset: intensity change of the CD signal at 645 nm as a function of the **Cy7** concentration. Conditions: [DNA] = 50 µM, [Co(III)] = 12 µM, [NaCl] = 10 mM, 5% MeOH + Na-cacodylate buffer (1mM, pH = 7.0). Titration step: [Cy7] = 2.0 µM.

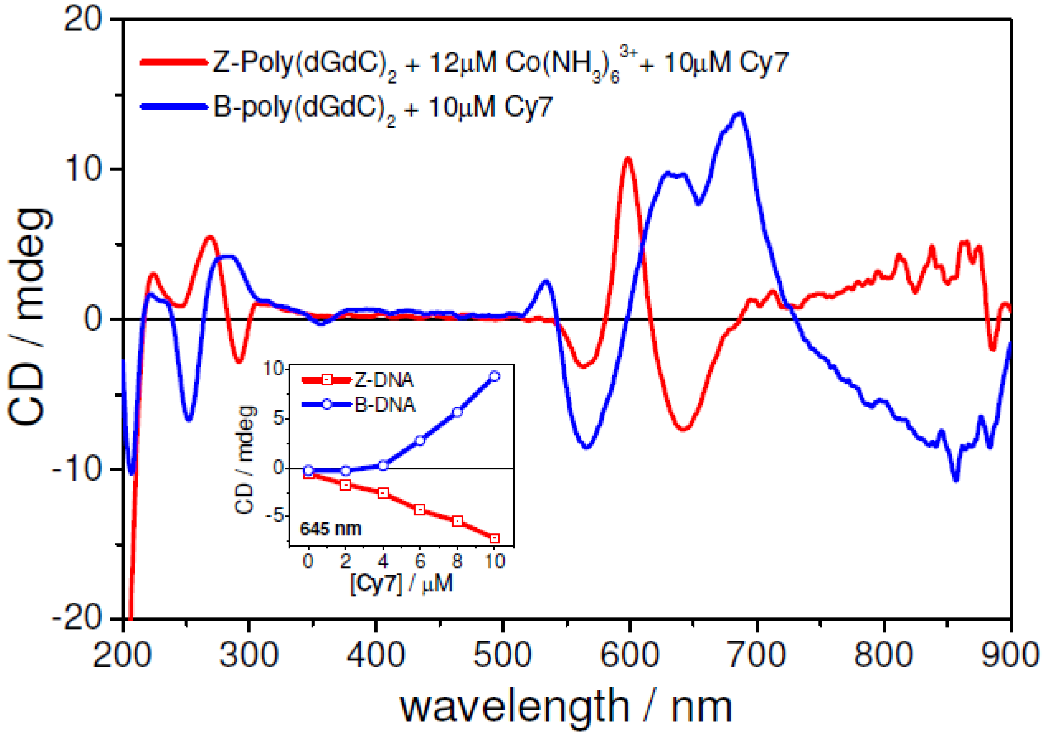

© 2011 by the authors; licensee MDPI, Basel, Switzerland. This article is an open access article distributed under the terms and conditions of the Creative Commons Attribution license (http://creativecommons.org/licenses/by/3.0/).
